# Evaluation of an exercise-enabling control interface for powered wheelchair users: a feasibility study with Duchenne muscular dystrophy

**DOI:** 10.1186/s12984-020-00760-9

**Published:** 2020-10-28

**Authors:** Joan Lobo-Prat, Aure Enkaoua, Antonio Rodríguez-Fernández, Nariman Sharifrazi, Julita Medina-Cantillo, Josep M. Font-Llagunes, Carme Torras, David J. Reinkensmeyer

**Affiliations:** 1 Institut de Robòtica i Informàtica Industrial, CSIC-UPC, Llorens i Artigas 4-6, 08028 Barcelona, Spain; 2 Biomechanical Engineering Lab, Department of Mechanical Engineering and Research Center for Biomedical Engineering, Universitat Politècnica de Catalunya, Diagonal 647, 08028 Barcelona, Spain; 3 Institut de Recerca Sant Joan de Déu, Santa Rosa 39-57, 08950 Esplugues de Llobregat, Spain; 4 Servei de Rehabilitació i Medicina Física, Hospital Universitari Sant Joan de Déu, Passeig de Sant Joan de Déu 2, 08950 Esplugues de Llobregat, Spain; 5 Department of Mechanical and Aerospace Engineering, University of California Irvine, Engineering Gateway 4200, Irvine, 92617 USA; 6 Departments of Anatomy and Neurobiology, Mechanical and Aerospace Engineering, Biomedical Engineering, and Physical Medicine and Rehabilitation, University of California Irvine, Engineering Gateway 4200, Irvine, 92617 USA

**Keywords:** Powered wheelchair, Physical exercise, Duchenne muscular dystrophy, Driving performance, Rare disease

## Abstract

**Background:**

Powered wheelchairs are an essential technology to support mobility, yet their use is associated with a high level of sedentarism that can have negative health effects for their users. People with Duchenne muscular dystrophy (DMD) start using a powered wheelchair in their early teens due to the loss of strength in their legs and arms. There is evidence that low-intensity exercise can help preserve the functional abilities of people with DMD, but options for exercise when sitting in a powered wheelchair are limited.

**Methods:**

In this paper, we present the design and the feasibility study of a new version of the MOVit device that allows powered-wheelchair users to exercise while driving the chair. Instead of using a joystick to drive the wheelchair, users move their arms through a cyclical motion using two powered, mobile arm supports that provide controller inputs to the chair. The feasibility study was carried out with a group of five individuals with DMD and five unimpaired individuals. Participants performed a series of driving tasks in a wheelchair simulator and on a real driving course with a standard joystick and with the MOVit 2.0 device.

**Results:**

We found that driving speed and accuracy were significantly lowered for both groups when driving with MOVit compared to the joystick, but the decreases were small (speed was 0.26 m/s less and maximum path error was 0.1 m greater). Driving with MOVit produced a significant increase in heart rate (7.5 bpm) compared to the joystick condition. Individuals with DMD reported a high level of satisfaction with their performance and comfort in using MOVit.

**Conclusions:**

These results show for the first time that individuals with DMD can easily transition to driving a powered wheelchair using cyclical arm motions, achieving a reasonable driving performance with a short period of training. Driving in this way elicits cardiopulmonary exercise at an intensity found previously to produce health-related benefits in DMD.

## Background

Improvements in health care have extended the life expectancy of people with neuromuscular disorders, and, as a result, many people with neuromuscular disorders make use of powered wheelchairs for a substantial part of their lives. Boys with Duchenne muscular dystrophy (DMD), for example, now have a life expectancy of 35 years and begin requiring a powered wheelchair around their early teens [1]. While powered wheelchairs are an essential technology to support mobility, their continuous use results in an increased level of sedentarism, which leads to secondary functional deterioration of the musculoskeletal and cardiorespiratory systems [2], as well as to an accelerated loss of arm function [3].

Current international guidelines for the management of DMD recommend regular submaximal activities that avoid eccentric or exhausting high-resistance exercises [4], yet there is no consensus on the specific exercise dose that should be given to people with neuromuscular disorders [5]. Jansen et al. [6] carried out a randomized control trial in which they evaluated the therapeutic effect of dynamic physical training in boys with DMD. Thirty boys (mean age 10.5 ± 2.6 years, 18 ambulant and 12 wheelchair-dependent) were randomly allocated to the intervention ($\hbox {n} = 17$) or the control group ($\hbox {n} = 13$). The intervention group received assisted bicycle training of the legs and arms over 6 months. The total Motor Function Measure score remained stable in the intervention group, but significantly decreased in the control group. Thus, an appropriate, long-term dose of dynamic physical training can help preserve the functional abilities of boys with DMD.

Current exercise devices for powered wheelchair users (such as the hand and leg cycles used in the aforementioned study [6]) require the user to drive their wheelchair up to the device, and then exercise during a fixed time period, which does not allow for integrated daily exercise. To address this limitation, we have previously developed MOVit, a novel exercise-enabling interface for powered wheelchairs [7]. The first version of MOVit consisted of two custom-made, spring-balanced, two degree-of-freedom (DOF), instrumented mobile arm supports that were mounted on the sides of a powered wheelchair replacing the arm rests. Instead of using a joystick to drive the wheelchair, the user moves the arm supports with their arms through a cyclical motion. We carried out a series of driving tests with a group of unimpaired individuals and showed for the first time the feasibility of exercising while driving a powered wheelchair [7].

In this paper we present an improved version of the MOVit device (MOVit 2.0) designed for individuals with DMD, and the results of a feasibility study carried out with five boys with DMD and five unimpaired individuals. All participants performed a series of driving tasks in a wheelchair simulator and on a real driving course with a standard wheelchair joystick and with the MOVit 2.0 device. The main objectives of this feasibility study were: (1) to determine if the group of boys with DMD could reach an acceptable driving performance while using MOVit 2.0 compared to the driving performance using a joystick, and (2) to evaluate the exercise intensity in terms of heart rate increase when using MOVit 2.0 compared to using a joystick.

## Materials and methods

### Experimental device: MOVit 2.0

**Fig. 1:**
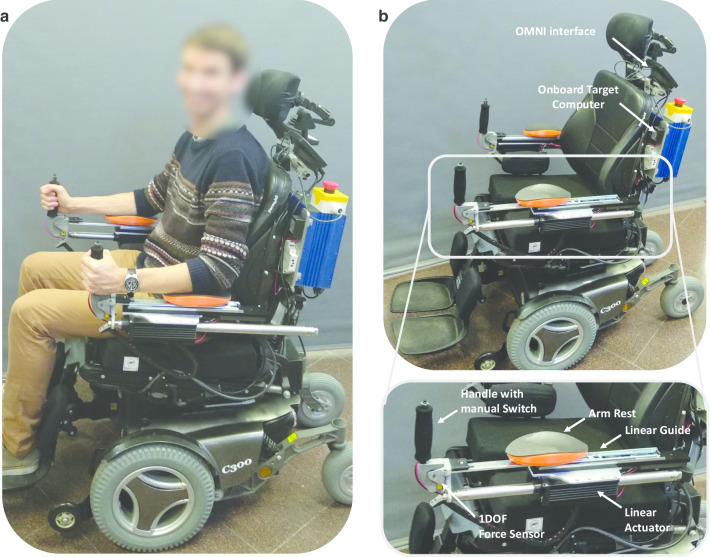
MOVit 2.0 Exercise-Enabling Powered Wheelchair. The device is composed of two single DOF, powered, mobile arm supports that replace the arm rests of the wheelchair. **a** An unimpaired individual using the MOVit 2.0 device. **b** Components of the MOVit 2.0 device with a detailed view of the MOVit Arm

**Fig. 2:**
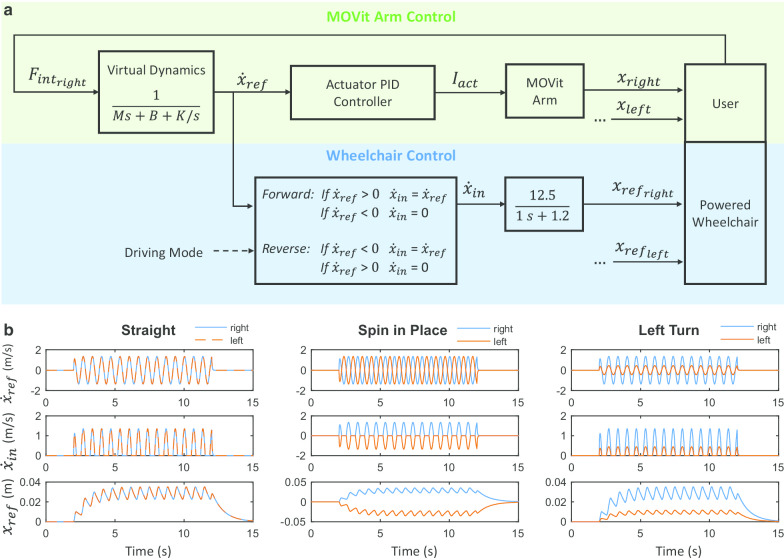
The control interface of MOVit 2.0. **a** The movement of the MOVit arms (green area) is controlled using the measured interaction force ($F_{int}$) as input of an admittance model that simulates the dynamics of a virtual mass-spring-damper system. The admittance model outputs a reference velocity (${\dot{x}}_{ref}$) that is sent to the low level PID controller of the actuator. The movement of the powered wheelchair (blue area) is controlled using the reference velocity ($\dot{x}_{ref}$) and the driving mode as inputs. The user can choose between the two driving modes (forward/reverse) by pressing the manual switch of the handle, which acts as a virtual clutch. In the forward mode, only the positive reference velocity will pass, and in the reverse mode, only the negative reference velocity will pass. The output ($\dot{x}_{in}$) is then filtered by a low pass filter that smooths out the input signal for the powered wheelchair ($x_{ref}$), which mimics the input signal of a 1 DOF joystick where the joystick displacement is proportional to speed. Note that each MOVit arm controls the velocity of its corresponding wheel and that for simplicity this diagram shows only the control diagram of the right side. **b** Illustrative signals of the reference velocity ($\dot{x}_{ref}$), output of the virtual clutch ($\dot{x}_{in}$), and reference position ($x_{ref}$, which is proportional to the wheel velocity) for straight driving, spinning in place, and left turning

The MOVit 2.0 device builds upon our previous work on developing an exercise-enabling driving interface for powered wheelchair users [7]. The main improvement of MOVit 2.0 is that it includes a linear actuator that allows the adjustment of the level of assistance/resistance that the device provides to the user’s arm movement (Fig. 1). Specifically, MOVit 2.0 consists of two powered, mobile arm supports that allow forward/backward motion of the arm along a telescopic linear guide actuated by a linear actuator (Servotube Actuator STA2504P, Dunkermotoren GmbH, Germany). The user interfaces with the device by resting his/her arms on an arm rest mounted on top of the linear guide and grasping the handle that is instrumented with a one DOF force sensor (LSB200, Futeck Inc., USA). The motion of the mobile arm supports of MOVit 2.0 are controlled using admittance control with virtual dynamics that simulate a mass-spring-damper system (see Fig. 2a). The device has a maximum stroke of 0.28 m and was mounted onto the arm rests of a Permobil c300 powered wheelchair (Permobil, Sweden).

Sensor signals and actuator commands were interfaced with a data acquisition card (NI PCI-6229, National Instruments Inc., USA), with a sampling frequency of 1 kHz and 16 bit resolution. The signal processing and control were programmed in Matlab Simulink 2016b running in a computer with Windows 10 Operating System and compiled to run on a Simulink Real-Time Target computer. The MOVit controller outputs the desired speed and heading of the powered wheelchair (Permobil c300, Permobil AB, Sweden) by sending two analog signals to the wheelchair controller through the R-Net Omni interface (PG Drives Technology, Curtiss-Wright Corp., USA). The target computer (I10 DDR4, Inctel Technology Co. Ltd, China), the force sensor amplifiers (IAA100, Futek Inc., USA), and the motor drivers (ADP-055-18, Copley Inc., USA) were mounted on the back of the wheelchair and powered directly from the wheelchair batteries.

As in the first version of MOVit, the control interface of MOVit 2.0 was designed to mimic the movement of propelling a manual wheelchair: each MOVit arm controls the movement of its corresponding wheel. In contrast to the first version of MOVit, the control interface of MOVit 2.0 does not require a clutch action. Instead, the user can choose to have the wheels in forward or reverse mode by pressing a manual switch that is located on top of the handles. This change was done with the intention to reduce the cognitive workload required by the clutch action. To go forward the user needs to move both arms in phase and at the same speed. To go backwards the user needs to press the switch of both sides (to enter the reverse mode) and move the arms in phase and at the same speed. To turn, the user needs to move one arm faster than the other, and to spin in place the user need to set one side in forward mode, the other side in reverse mode and move the arms with a phase shift of 180 degrees between them. The details of the control interface are described in Fig. 2. The parameters of the admittance model were set to $\textit{M} = 5\,\hbox {kg}$, $\textit{B} = 10\,\hbox {Ns/m}$, and $\textit{K} = 100\,\hbox {N/m}$ for the unimpaired participants and $\textit{K} = 20\,\hbox {N/m}$ for the participants with DMD. Stiffness values were set lower for participants with DMD to prevent excessive fatigue.

### Participants

A total of five unimpaired males (mean age: 25 ± 1.5 years) and seven males with DMD participated in this feasibility study. Note that DMD primarily affects males. The data files of one participant with DMD were found to be corrupted, and another participant with DMD was not able to complete the experimental protocol due to a high level of anxiety when testing the MOVit device. Thus, we report here the results of five participants with DMD (see Table 1). All participants provided informed consent to participate in this experiment, which was approved by the Ethical Committee of Fundació Sant Joan de Déu (Barcelona, Spain; study code: PIC-83-19 / PS-03-19).

**Table 1:** Characteristics of the participants with DMD

Subject code	Age (years)	Brooke scale	Resting heart rate (bmp)	Powered wheelchair user
S1	13	2	98.2	Yes
S2	16	3	79.3	Yes
S3	17	2	94.1	Yes
S4	14	3	130.6	Yes
S5	15	4	95.5	Yes

### Experimental tasks and protocol

**Fig. 3:**
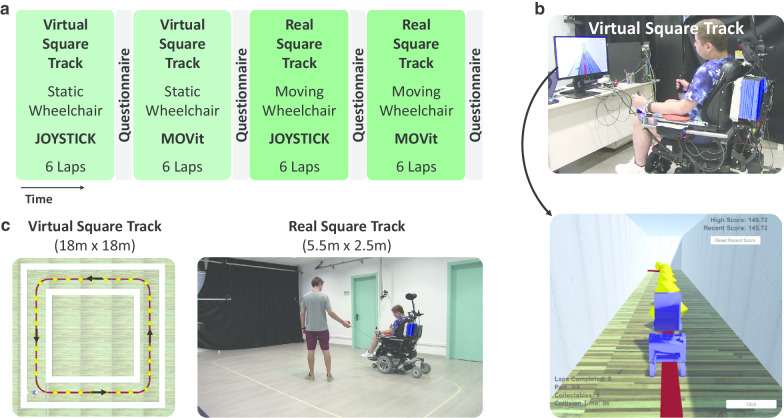
Experimental tasks and protocol. **a** Timeline of the experimental protocol, which included driving six laps in a wheelchair simulator and six laps in the real driving course with a standard joystick and with the MOVit 2.0 device. **b** A participant with DMD performing the driving task in the wheelchair simulator. **c** Pictures of the Virtual Square Track and the Real Square Track. Both tracks had a line on the floor that indicated the optimal path.

The experimental protocol was carried out in a single session that had a duration of approximately 1.5 h (Fig. 3a). First, participants were asked to drive six laps of a virtual square track in a wheelchair simulator using first a joystick, and afterwards using the MOVit 2.0 device (Fig. 3b). Subsequently, participants were asked to repeat the same task in a real square track (Fig. 3c). The joystick of an Xbox wireless controller was used for the Virtual Driving Task, while the wheelchair joystick was used for the Real Driving Task. We instructed participants to “follow the line on the floor and drive as fast as possible”. Before each task, participants had to practice the driving course a minimum of 3 laps and a maximum of 6 laps. In addition, after completing each of the driving tasks, the participants with DMD had to answer a series of questions to evaluate their driving experience (Table 2). Participants rested for 5 to 10 min after completing each of the driving tasks. For both control methods and both driving tasks the maximum wheelchair velocity was set to 0.75 m/s.

**Table 2:** Questionnaire

Rate from 0 (I disagree) to 10 (I agree)
1. After working at this activity for a while, I felt pretty competent
2. I am satisfied with my performance at this task
3. This activity was hard to do
4. I felt nervous doing this activity
5. I felt fatigued during the task
6. I felt comfortable using the MOVit device
7. I feel that my muscles are sore
Answer with Yes/No
8. I find using MOVit more fun than using a Joystick
9. I would like to use MOVit during my daily life

### Data analysis

Driving performance was evaluated in terms of speed, path root-mean-square error (RMSE), maximum path error, and path smoothness, which was calculated using the Spectral Arc Length (SPARC) of the wheelchair velocity [8]. Arm movement performance was evaluated in terms of arm movement amplitude, arm movement frequency, arm movement synchrony, and interaction force. Arm movement synchrony was measured calculating the cross-correlation coefficients of the left and right arm movement signals, and interaction force was calculated as the average peak force for each arm movement repetition. Finally, exercise intensity was evaluated in terms of heart rate increase, which was measured using a wearable chest strap sensor (Polar H10, Polar Electro Oy, Finland). Resting heart rate was measured at the beginning of the experiment by asking the participant to relax for five minutes and calculating the average value over the last minute. Heart rate increase was calculated taking the average heart rate value during a driving task and subtracting the resting heart rate from it.

All the driving performance metrics were calculated from the path data of the wheelchair simulator for the virtual driving tasks. For the real driving tasks, the path data was measured using a motion capture system with nine cameras (Optitrack Flex 3, NaturalPoint Inc., USA) and two reflective markers mounted on the head rest of the powered wheelchair.

To compare the performance metrics (*Score*) of the two *Groups* of participants (Unimpaired and DMD) with the two control *Inputs* (joystick and MOVit 2.0), a linear mixed-effects analysis was conducted on all metrics for each driving task (i.e., virtual and real driving tasks). For the driving performance metrics, we modeled *Input* and *Group* (and their interaction) as fixed effects, and used an error term with random intercepts grouped by *Subject* (Eq. 1). For the arm movement performance metrics, we modeled *Group* as fixed effect, and used an error term with random intercepts grouped by *Subject* (Eq. 2). Analysis of variance (ANOVA) tests were used to compare the performance scores for each of the performance metrics, and Bonferroni tests were applied for pairwise comparison. Statistical analysis of the questionnaire results was performed with paired t-tests. We used $\alpha = 0.05$ as the level of significance. Statistical analyses were carried out using R 3.5.0 [9] with lme4: Fitting Linear Mixed-Effects Models [10], lmerTest: Tests in Linear Mixed-Effects Models [11], and lsmeans: Least-Squares Means [12].

1 $$\begin{aligned}&Score \sim Group * Input + (1|Subject) \end{aligned}$$

2 $$\begin{aligned}&Score \sim Group + (1|Subject) \end{aligned}$$

## Results

**Fig. 4:**
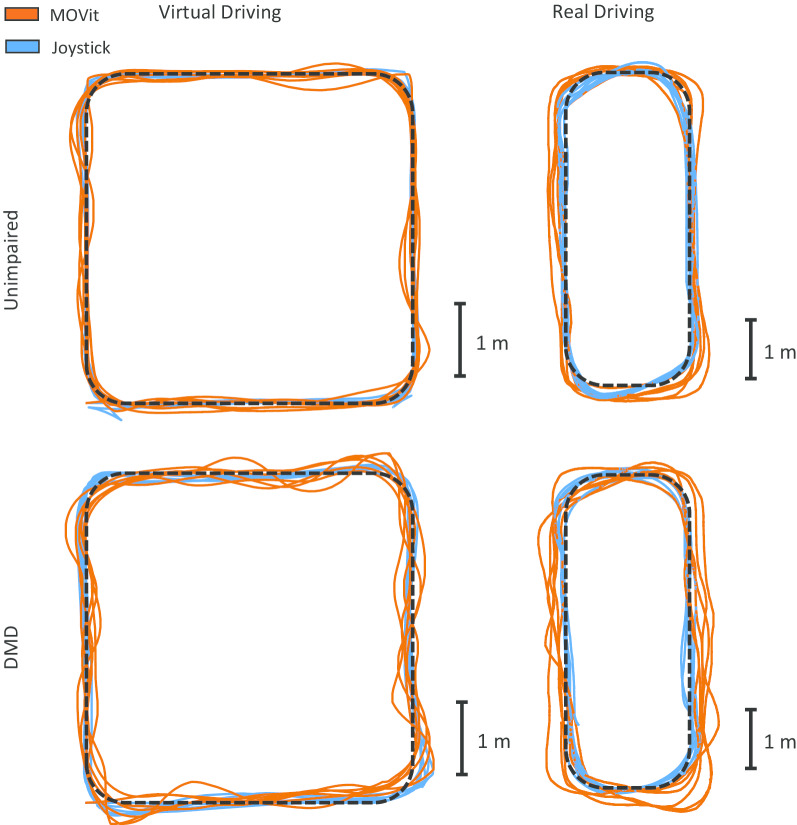
Illustrative wheelchair paths. Wheelchair paths of an unimpaired subject and a subject with DMD performing the virtual driving and real driving tasks, using the standard joystick (blue) and the MOVit 2.0 device (orange). Note that paths performed by the subject with DMD are slightly more irregular

The five DMD participants were aged 13–17 years and had a Brooke scale ranging from 2–4 (see Table 1), where a Brooke score of 2 indicates moderate reduction in the ability to raise the hands over the head, and a score of 4 indicates an inability to raise an 8 oz (227 g) of water to the mouth. All DMD participants were powered wheelchair users. Both the DMD and unimpaired participants were able to successfully complete all the driving tasks. Additional file 1 is a video that shows a participant with DMD performing the virtual and real driving tasks using the joystick and the MOVit control inputs. Figure 4 shows six wheelchair paths from an unimpaired participant and six wheelchair paths from a participant with DMD when driving the Virtual Square Track and the Real Square Track with a standard joystick and with the MOVit 2.0 device. In the next section we quantify the differences between the two control methods and the two groups of participants.

**Fig. 5:**
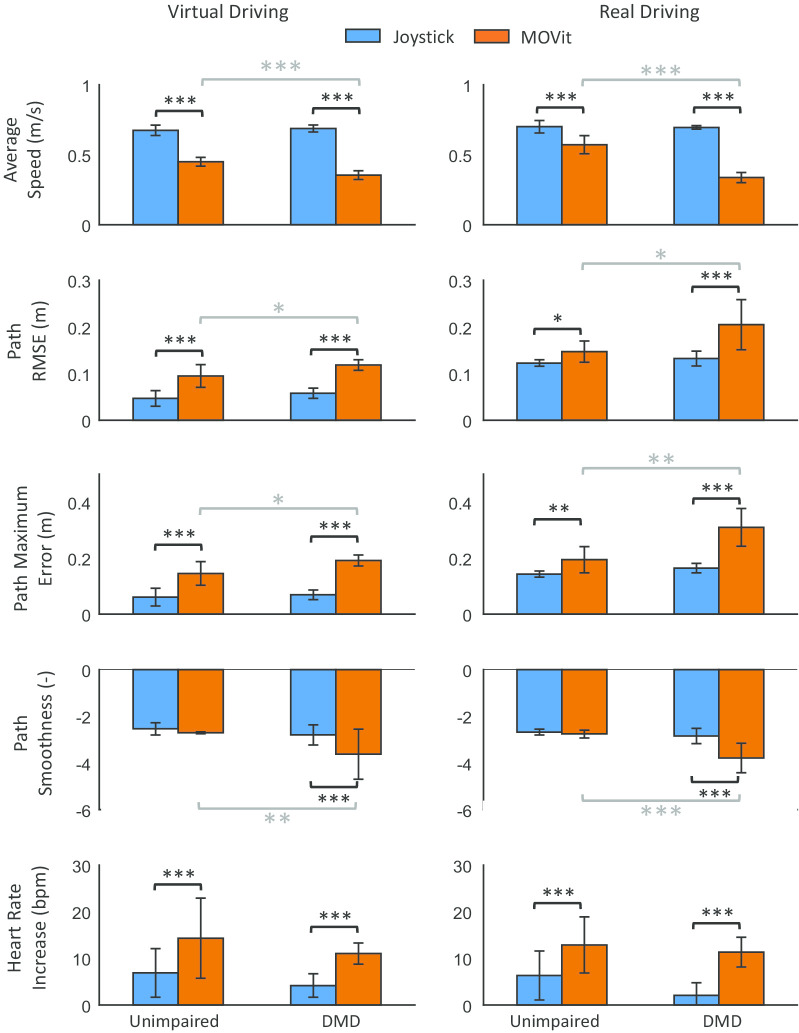
Wheelchair-driving performance evaluation. Shown are the driving performance metrics for the unimpaired participants and the participants with DMD when performing the virtual and real driving tasks with the joystick (blue) and MOVit (orange). Bar height indicates the average value across participants and the error bar indicates one standard deviation. Number of asterisks indicates the level of significance: *$p<0.05$, **$p<0.005$, and ***$p<0.001$

### Wheelchair-driving performance evaluation

We found significant differences for all driving performance metrics when comparing the two control inputs (i.e., joystick vs. MOVit; Fig. 5). Compared to driving with the joystick, driving with MOVit significantly decreased speed in both groups and tasks by an average 0.26 m/s (37.7% reduction). Driving speed when using the joystick did not significantly differ between groups ($p>0.05$), yet participants with DMD were significantly slower (virtual driving task mean diff.: 0.096 m/s, real driving task mean diff.: 0.24 m/s, $p<0.001$) than unimpaired participants when using MOVit for both tasks.

Regarding the path RMSE, we found that MOVit significantly increased the path errors for both groups and driving tasks by an average 0.05 m, compared to the joystick. These path errors were significantly larger for the participants with DMD (virtual driving task mean diff.: 0.02 m, real driving task mean diff.: 0.06 m, $p<0.05$). In contrast, path errors when using the joystick were not significantly different between groups ($p>0.05$).

Similar to the results of the path RMSE, path maximum errors were also significantly larger when using MOVit for both groups and driving tasks by an average 0.1 m. Participants with DMD had significantly larger path maximum errors compared to unimpaired individuals (virtual driving task mean diff: 0.05 m, $p<0.05$; real driving task mean diff.: 0.12 m, $p<0.005$).

Path smoothness in both driving tasks was significantly lower by an average 0.9 ($p<0.001$) for participants with DMD when using MOVit compared to the joystick. We also found that participants with DMD had a significantly lower smoothness (virtual driving mean diff.: 0.91, $p<0.005$; real driving mean diff.: 1.03, $p<0.001$) than unimpaired participants when using MOVit for both driving tasks. No significant differences were found for unimpaired participants.

Finally, compared to when using the joystick, we found that MOVit led to a larger heart rate increase in both groups and tasks by an average 7.5 bpm ($p<0.001$). Heart rate increase did not significantly differ between groups.

**Fig. 6:**
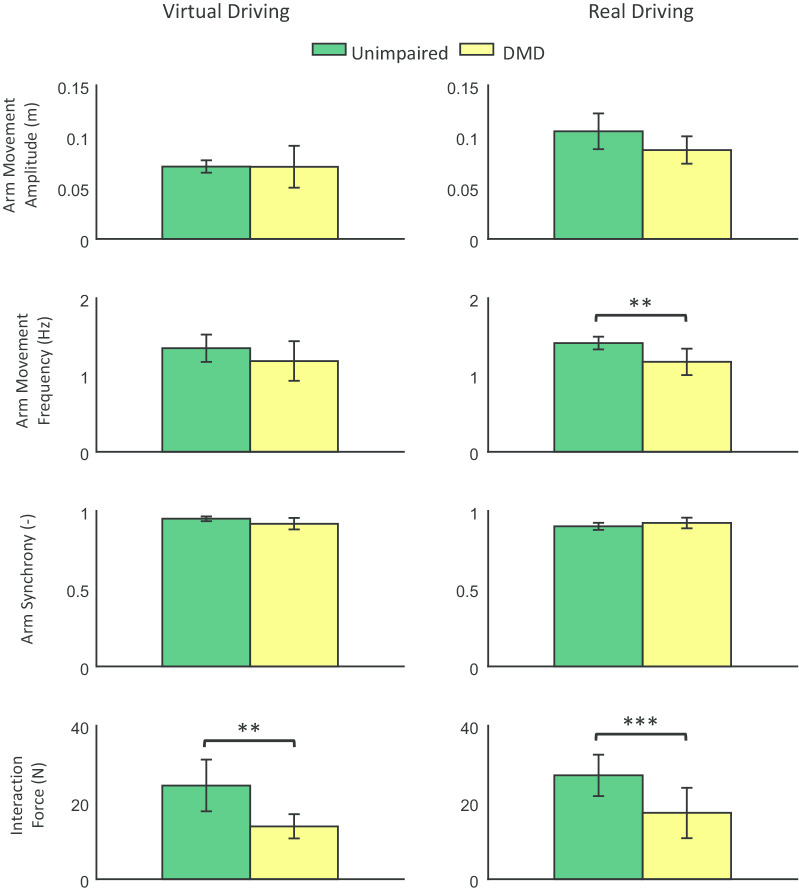
Arm movement performance evaluation. Bar plots of the arm movement performance metrics for the unimpaired participants (green) and the participants with DMD (yellow) when performing the virtual and real driving tasks with MOVit. Bar height indicates the average value across participants and the error bar indicates one standard deviation. Number of asterisks indicates the level of significance: *$p<0.05$, **$p<0.005$, and ***$p<0.001$

#### Arm movement performance evaluation

Figure 6 shows the results of the arm movement performance metrics. Generally, participants with DMD had similar arm movement features compared to the unimpaired participants, although two measures differed significantly. When using MOVit in the real driving task, participants with DMD had a significantly lower arm movement frequency (mean diff.: 0.2 Hz, $p<0.005$) and lower interaction force (virtual driving mean diff.: 10.7 N, $p<0.005$; real driving mean diff.: 9.8 N, $p<0.001$) compared to the unimpaired participants. Note that the reduction in interaction force was expected as it is a direct consequence of using different virtual spring stiffnesses for participants with DMD ($\textit{K} = 20\,\hbox {N/m}$) and unimpaired participants ($\textit{K} = 100\,\hbox {N/m}$). Arm movement amplitude and arm synchrony did not significantly differ between groups.

#### Questionnaire results

**Fig. 7:**
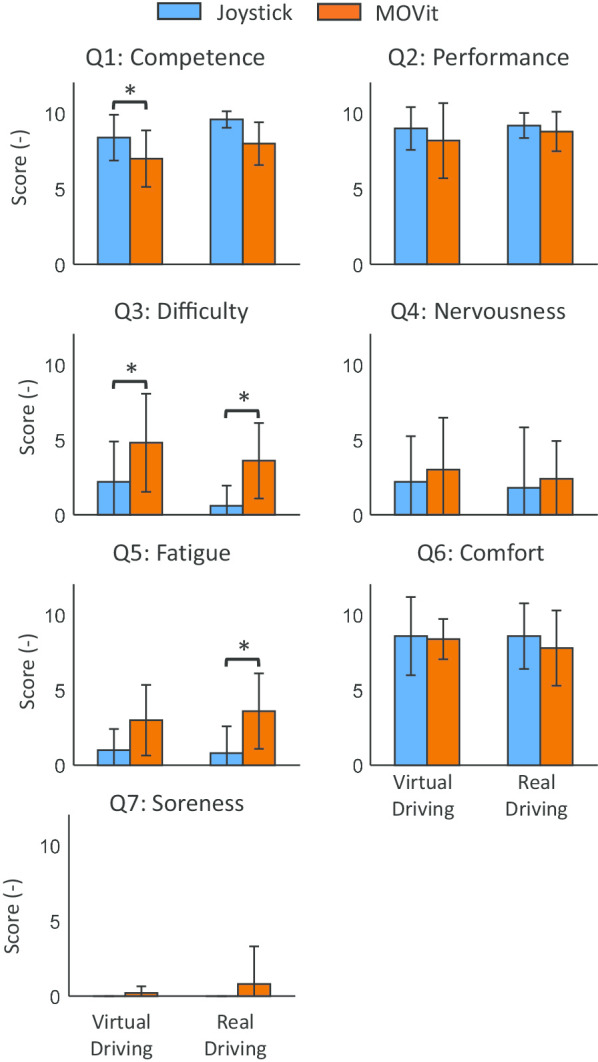
Questionnaire results. Average and standard deviation values of the scores provided by the participants with DMD when answering the questionnaire after performing the virtual and real driving tasks with the joystick (blue) and MOVit (orange). Number of asterisks indicates the level of significance: *$p<0.05$

Figure 7 shows the results of the Questionnaire. We found that during the virtual driving tasks, participants with DMD felt significantly more competent when driving with the joystick than with MOVit, although the difference was small (mean diff.: 1.4 on a 10 point scale, $p<0.05$). In addition, participants with DMD reported that driving with MOVit was significantly more difficult than using the joystick when performing both driving tasks (virtual driving mean diff.: 2.6; real driving mean diff.: 3, $p<0.05$). Finally, when performing the real driving task, participants with DMD felt significantly more fatigued when using MOVit than when using the joystick (mean diff.: 2, $p<0.05$). Questions regarding satisfaction with performance and comfort while using the joystick or MOVit, as well as nervousness and soreness, did not show a significant difference between the two control inputs.

All participants with DMD agreed that driving the wheelchair with MOVit was more fun than using the standard joystick. In addition, three out of the five participants responded that they would like to use MOVit regularly during daily life, and the other two participants mentioned that they would use MOVit for exercising but not as their regular control interface.

## Discussion

This study shows for the first time that individuals with DMD can quickly learn to drive a powered wheelchair using cyclical arm motions, achieving a reasonable driving performance with which they felt subjectively satisfied and comfortable. Driving with cyclical arm motions also achieved the exercise effect that we intended—a significant increase in heart rate. We first discuss their driving performance, then the level of exercise they achieved, followed by a description of the study limitations and envisaged directions for future research.

### Evaluation of driving performance

To evaluate if the group of participants with DMD could reach an acceptable driving performance while using MOVit 2.0, we compared it to their driving performance when using a joystick. While our results indicate that DMD patients performed significantly better with the joystick than with MOVit 2.0, these differences (mean speed diff: 0.26 m/s and maximum path error: 0.1 m) are probably inconsequential in terms of achieving daily life activities. Considering that the participants with DMD were well-experienced with joystick control, while they had only a relatively short training experience with MOVit 2.0, it is likely that this difference would be reduced with more training.

The study design also allowed us to compare the driving performance between participants with DMD and unimpaired participants. With a joystick, their driving performance was comparable. However, when using MOVit 2.0, unimpaired participants presented a better driving performance than participants with DMD in terms of both speed and path accuracy, especially when they were tested in the real driving environment. The observed increase in speed was due to the fact that unimpaired participants chose a significantly higher value of arm movement frequency (mean diff: 0.2 Hz), which may have been possible because they had greater strength and/or better motor control of the arms than the participants with DMD. With further training, the individuals with DMD may learn to drive faster. It may also be possible to better tune the driving controller, including the mass-spring-damper parameters and how arm movement frequency and amplitude map to wheelchair speed.

### Exercising with cyclical arm movements

The underlying motivation of the MOVit system is to provide arm exercise sufficient to improve fitness, and therefore health and quality of life for powered wheelchair users. The fact that frequent light to moderate physical activity can result in significant health-related benefits for unimpaired sedentary people is a well-studied phenomenon [13]. Physical exercise has also been shown to reduce secondary health problems in patients with different kinds of neuromuscular disorders such as stroke, spinal cord injury and multiple sclerosis [14–16]. For the case of people with DMD and other types of neuromuscular disorders, the therapeutic community is still debating the optimal exercise dose required to benefit fitness and health. In one of the few studies on this aspect, Jansen et al. [6] showed that an appropriate, long-term dose of dynamic physical training can help preserve the functional abilities of boys with DMD. Noteworthy, the level of physical training advised by Jansen et al. [6] was comparable to the one measured in the present study: participants in [6] were instructed to perform assisted bicycle training for 15 min with both their legs and arms, turning the cycle at 65 rpm, a rate that corresponds to 1.08 Hz. On average, participants with DMD in the present study performed arm movements at 1.2 Hz. As a result of this cyclical arm movement, heart rate increased for both unimpaired and DMD participants when using MOVit 2.0. The participants with DMD also reported a significantly higher fatigue level than the unimpaired participants, while also reporting a low level of muscle soreness and a high level of comfort in using the device.

Another positive consequence of light exercise for people with DMD might be lowering their resting heart rate, as occurs for unimpaired individuals [17]. This effect has not been yet studied in humans with DMD, but results of studies using DMD mouse models have shown that voluntary exercise is beneficial to the skeletal muscle and heart function, and does not aggravate the muscle pathology [18]. The beneficial effect of light exercise on lowering resting heart rate in people with DMD is thus a reasonable hypothesis. Furthermore, considering that the resting heart rate of boys with DMD is higher than that of unimpaired participants [19, 20], and that their heart rate increases with age up to the onset of cardiomyopathy instead of the normal age-related decline [19], the beneficial effects of light exercise might actually be relatively higher and more relevant for people with DMD than for people without impairment. Therefore, the use of MOVit 2.0 to promote physical exercise could help DMD patients not only by preserving their functional abilities, but also by preventing and delaying the onset of cardiac complications.

### Limitations and future work

The conclusions of this study need to be regarded with caution due to the low number of participants. The access to suitable subjects is limited due to the low density of people with DMD (i.e. 1:5000 male newborns [21]) and the legal and ethical constraint that they can only participate in one study at the same time. In the allowed time window of this study, we had access to seven participants that met all criteria and were able and willing to participate. Since the main goal of this study was to investigate the feasibility of the MOVit device, we performed the tests with these seven participants (with data from five of them included in the analysis), which allowed for an exploratory assessment of the MOVit device. Therefore, our results indicate, but cannot demonstrate at population level, the feasibility of the MOVit device for boys with DMD as an exercise-enabling interface for driving their powered wheelchairs.

In this study we took the first step of evaluating the use of MOVit 2.0 by individuals with DMD in a single training session in a controlled laboratory setting. This leaves open three key questions: (1) How skilled can persons with DMD become at driving MOVit 2.0 with extended training?; (2) Can their skill level become high enough so that they are safe and comfortable with driving in the real world?; and (3) Is the type of arm exercise that is possible with MOVit sufficient to produce long-term health effects? To answer these questions, future work will test long-term use of MOVit in the real world by individuals with DMD.

Because MOVit is computer-controlled, it will be possible to allow the user to select periods of time to use MOVit for exercise while driving, but also to revert to using MOVit in a joystick-like control mode when desired. Joystick-like control can be achieved by changing the controller to require only small motions of each arm to drive the chair, removing the requirement of large cyclical arm movements. To provide another option for exercise, we are also working on developing an interactive gaming interface for MOVit. The user can activate this interface when he pulls up to a gaming console or computer, or to control games on a phone or tablet placed on his lap. Since MOVit can measure and record the amount of arm exercise achieved throughout the day, it will be possible for the system to provide feedback and make recommendations for use of these various exercise strategies to achieve a daily target amount of exercise.

Another interesting direction for future research is optimizing the control strategy that specifies both the arm exercise profile and the driving method. Here, we provided light resistance to the users with a simulated virtual mass-spring-damper system. Adults with DMD typically have greater arm weakness than the adolescents tested here, and thus implementing control strategies based on providing movement assistance may be helpful. The fact that it is possible to experiment with different controllers and exercise profiles emphasizes the fact that MOVit 2.0 provides a flexible and powerful platform to optimize and understand how various forms of arm exercise can improve fitness and health in DMD and other conditions.

## Conclusions

This paper presented the design and testing of MOVit 2.0, building upon our previous work in developing an exercise-enabling driving interface for powered wheelchair users. Here we tested the improved interface for the first time with individuals with neuromuscular impairments. Results of this feasibility study revealed that participants with DMD were able to quickly learn to use cyclical arm motions to drive a powered wheelchair with reasonable driving performance and comfort with the system. While participants performed significantly better with the joystick than with MOVit, these differences would probably be inconsequential in terms of altering daily life activities and most likely can be reduced with further training. Using MOVit caused light arm exercise at a level that has been shown to produce health-related benefits for people with DMD. The experience with MOVit 2.0 was positively assessed by participants with DMD, and the majority of participants were interested in using the system regularly during daily life. In conclusion, we have shown for the first time the feasibility of a control interface that can be used by people with physical impairment as a means to exercise while driving a powered wheelchair.

## Supplementary information

**Additional file 1.** Video of a participant performing the driving tasks. This video shows a participant with DMD performing the virtual and real driving tasks using the joystick and the MOVit control inputs.

## Data Availability

The datasets used and/or analysed during the current study are available from the corresponding author on reasonable request.

## Abbreviations

DMD: Duchenne muscular dystrophy
DOF: degree-of-freedom
SPARC: Spectral Arc Length
RMSE: root-mean-square error
